# Epidemiology of comorbid hazardous alcohol use and insomnia in 19 185 women and men attending the population-based Tromsø Study 2015–2016

**DOI:** 10.1186/s12889-022-13250-5

**Published:** 2022-04-27

**Authors:** Vendela H. Husberg, Laila A. Hopstock, Oddgeir Friborg, Jan H. Rosenvinge, Svein Bergvik, Kamilla Rognmo

**Affiliations:** 1 Department of Psychology, Faculty of Health Sciences, UiT The Arctic University of Norway, Tromsø, Norway; 2 Department of Community Medicine, Faculty of Health Sciences, UiT The Arctic University of Norway, Tromsø, Norway

**Keywords:** Hazardous alcohol use, Insomnia, Population-based study, AUDIT

## Abstract

**Background:**

Hazardous alcohol use is known to be comorbid with insomnia problems. The present study examined the prevalence of insomnia and if the odds of insomnia differed between women and men with a hazardous alcohol use.

**Methods:**

Cross-sectional data from the seventh survey of the Norwegian population-based Tromsø Study 2015–2016 (participation 65%). The sample included 19 185 women and men 40–96 years. Hazardous alcohol use was defined by the Alcohol Use Disorder Identification Test (AUDIT) and insomnia by the Bergen Insomnia Scale. Covariates included socio-demographics, shift work, somatic conditions and mental distress defined by Hopkins Symptom Check List-10 (HSCL-10). Mental distress was also included as a moderator.

**Results:**

Insomnia was more prevalent among participants with a hazardous alcohol use (24.1%) than without (18.9%), and participants who had hazardous alcohol use had higher odds of insomnia (odds ratio = 1.49, 95% CI = 1.20, 1.85). The association turned non-significant after adjustment for mental distress. Adding mental distress as a moderator variable revealed a higher odds of insomnia among hazardous alcohol users having no or low-to-medium levels of mental distress, but not among participants with high levels of mental distress.

**Conclusion:**

Insomnia was more prevalent among women and men reporting hazardous alcohol use. When mental distress was treated as a moderator, hazardous alcohol use did not yield higher odds for insomnia among those with high levels of mental distress. This suggests that mental distress may play an important role in the association between hazardous alcohol use and insomnia. And that the impact of alcohol on insomnia may differ depending on the severity of mental distress.

**Supplementary Information:**

The online version contains supplementary material available at 10.1186/s12889-022-13250-5.

## Background

Insomnia is the most common sleep disorder in the adult general population [1]. It occurs also highly comorbid with hazardous alcohol use and alcohol use disorders [2–4] with comorbid prevalence ranging between 7–52% in population-based samples [5, 6]. There are some well-known gender differences in the prevalence for both insomnia and hazardous alcohol use; women have more often insomnia [7, 8] while men use alcohol more hazardously [9, 10]. Several factors correlate with hazardous alcohol use and with insomnia, such as somatic and mental health conditions [11–14], older age, low socioeconomic status, or shift working [7, 14–16]. A problem, however, is the large variation in the reported general population prevalence estimates of co-occurring hazardous alcohol use and insomnia [5, 6, 17–22]. Methodological differences between the studies may be a contributor, e.g., large variations in gender distributions, which often are dominated by men [6, 17, 18], use of unrepresentative samples that increases the reported range, such as military veterans [17] and industrial workers [6], or variation in sample sizes including many small sample-sized studies [17, 19]. Also, contributing to the heterogeneity is the wide variations in the operationalization of hazardous alcohol use [5, 17, 23] and insomnia [17, 18, 21] across studies. In addition, studies vary in whether they adjust the prevalence estimates for comorbid mental health conditions [5, 17, 21]. We suggest that methodological improvements can be achieved by using population-based data, including a large representative sample of both women and men, and applying standardized, widely used and acceptable scales for the measure of hazardous alcohol use and insomnia and mental distress is necessary for estimating the comorbid prevalence of hazardous alcohol use and insomnia.

The primary aim of the present study was to estimate the gender-specific prevalence of comorbid hazardous alcohol use and insomnia in a representative population-based sample of women and men. Secondary, the aim was to estimate the association between hazardous alcohol use and insomnia, and to investigate the potential moderating role of mental distress in this association.

## Methods

### Sample and data collection

The Tromsø Study [24] is a population-based study with seven repeated surveys between 1974 and 2016 (Tromsø1-Tromsø7), inviting total birth cohorts and random samples of inhabitants in the municipality of Tromsø, Norway. The present study is based on data from Tromsø7. Data collection include questionnaires, biological sampling and clinical examinations. All registered inhabitants aged ≥ 40 years (*N* = 32 951) were invited to participate in Tromsø7 (2015–2016). The invitation included a personal letter with username and password for completion of online questionnaires before attendance. In total, 21 083 (65%) women and men attended.

Due to the listwise deletion criteria, the sample included in the various analyses varies somewhat in size. Data were available from between 19 185 and the full sample in the descriptive analyses, whereas data from 18 898 were included in the prevalence analyses. Data from 16 529 participants were available for inclusion in the multivariate analyses.

### Measures

#### Hazardous alcohol use

Hazardous alcohol use is defined as a pattern of alcohol consumption that increases the risk of harmful consequences for the user or others [9]. Alcohol consumption and hazardous drinking were measured with The Alcohol Use Disorder Identification Test (AUDIT) [25], an extensively validated and commonly used international screening instrument to identify hazardous drinking in the past year [26]. AUDIT consists of 10 items (score range 0–4) measuring alcohol consumption (frequency of drinking, amounts consumed when drinking, and frequency of binge drinking), behavior patterns (e.g., not being able to stop, need a drink in the morning) and consequences (e.g. failed expectations, feelings of guilt or remorse). AUDIT has a sum score range of 0 – 40, and in accordance with established practice, we used values ≥ 8 as a cut-off indicative of hazardous alcohol use [27]. Dichotomizing the summative scale at this cutoff is strong with a high degree of sensitivity (92%) and specificity (94%) for detecting problematic drinking/hazardous alcohol use [27]. We use the term “hazardous alcohol use” from here on, and may include individuals with more serious alcohol problems, e.g., alcohol dependency disorder.

#### Insomnia

Insomnia was measured by the Bergen Insomnia Scale (BIS) which has shown to have good psychometric properties. [22]. BIS consists of six items, including nocturnal symptoms (sleep onset latency, sleep maintenance, early morning awakening) and non-restorative sleep, daytime impairment and dissatisfaction with sleep, in the past four weeks. Response categories ranged from 0 = no days per week to 7 = 7 days per week. An additional item about the duration of sleep problems was included, with response options ranging from “do not have a sleeping problem” to “more than 10 years”. Insomnia was categorized as following: ≥ 3 days on at least one of the nocturnal symptoms, and ≥ 3 days per week on daytime impairment or dissatisfaction with sleep, and ≥ 3 months duration of sleep problems [28, 29], in accordance with DSM-5 and ICD-10 criteria of insomnia [30, 31].

#### Comorbidity

Somatic disease was defined as reporting at least one of the following (0-no, 1-yes): myocardial infarction (in the past), stroke (in the past), heart failure, atrial fibrillation, angina pectoris, hypertension, diabetes, cancer, kidney disease, chronic obstructive pulmonary disease, asthma, rheumatoid arthritis, arthrosis or migraine, in the past or present.

In addition, participants reported how often they had used sleep, anxiolytic and anti-depressant medication in the past four weeks.

Mental distress was measured by the Hopkins Symptoms checklist-10 (HSCL-10) [32]. It includes symptoms of anxiety (4 items) and depression (6 items), as occurring during the past week. The HSCL-10 is a widely used, well validated instrument [33]. Response categories ranged from 1 = no complaint to 4 = very much, and scores were averaged across all items. The 10 item version of HSCL is a short form of the HSCL-25, and performs almost equally well as the full version when measuring mental health [33]. HSCL-10 had a high internal consistency (Cronbach’s *α* = 0.87) in the current study.

#### Sociodemographic and socioeconomic factors

Age, educational level (primary school, upper secondary education, university education < 4 years, tertiary education > 4 years), living with spouse (yes/no), and shift work (yes/no) were included to adjust for sociodemographic and socioeconomic factors.

### Statistical analyses

To estimate the prevalence and confidence intervals (CIs) of insomnia among women and men, we used a two-sample test of proportions. In addition, we specified a logistic binomial regression model with insomnia (0-no, 1-yes) as the outcome variable, and hazardous alcohol use (0-no, 1-yes) as the predictor. Gender differences were modeled by adding the interaction term, hazardous alcohol use × gender. Effect sizes for the model parameters are given as odds ratios (OR), including 95% confidence intervals. In order to compare models, we specified a series of four nested logistic regression models: 1) model 1 included hazardous alcohol use and gender, in addition to the alcohol × gender interaction term in order to examine if the OR of insomnia was different for women and men, 2) in model 2 the variables age, education, living with spouse, and shift working were added, 3) in model 3 somatic disease, use of sleep, -anxiolytic and –antidepressant medication were added as covariates, and 4) in model 4 mental distress was added as a covariate. These analyses were performed in STATA 16 (STATA Corp LP Texas, USA). We additionally examined if mental distress moderated the relationship between hazardous alcohol use and insomnia using the PROCESS Macro in SPSS, developed by Hayes [34]. The PROCESS macro accepts modeling of binary outcomes through a log link function in order to estimate a linear beta parameter. Results are presented as odds ratios by retransforming log odds of beta to odds ratios (OR = e^beta log odds^). All covariates from model 4 in the nested regression analysis were retained. Mental distress was mean centered. Moderation analyses using PROCESS is a conditional process analysis, which means that it produces regression coefficients for the predictor-outcome relationship depending on the chosen moderator values. Thus, the regression coefficient of hazardous alcohol use is interpreted as the effect of a one unit increase on hazardous alcohol use (i.e. difference between individuals with and without a hazardous alcohol use) on log odds of insomnia when mental distress is 0, which after mean centering means average mental distress. Likewise, the regression coefficient of mental distress must be interpreted as the effect of a one unit increase in mental distress on log odds of insomnia when hazardous alcohol use is 0, or non-hazardous alcohol use. The moderation coefficient is interpreted as the change in the simple regression coefficient describing the association between hazardous alcohol use and insomnia as mental distress changes by one unit. The moderation effect was probed by applying cut-off scores at the 16^th^, 50^th^ and 84^th^ percentiles of mental distress. Johnson-Neyman region(s) of significance were reported, which identifies where along the mental distress score continuum (the moderator) the effect of hazardous alcohol use on insomnia turns from non-significant to significant at the chosen α-level (*p* = 0.05).

### Treatment of missing values

To reduce the risk of bias, missing values on the individual items of hazardous alcohol use, mental distress and insomnia were imputed using the Missing Values Analysis (MVA), Expectation Maximization (EM) method in SPSS version 25. For a missing value to be imputed, the record needed at least 50% valid data on the items of the instrument being imputed, the valid responses were used to impute the missing values. Thus, missing cases were reduced from 15.8% to 7.0% for hazardous alcohol use, from 6.0% to 3.3% for mental distress, and from 10.2% to 7.0% for insomnia.

To test for meaningful differences between the 16 529 participants included in the multivariate analysis, and the full sample (*n* = 21 083) *t*-tests and x^2^ tests were run on hazardous alcohol use, insomnia, mental distress, sex and age. A binary variable was created which distinguished between included and excluded participants. These tests were run on the unimputed versions of the variables.

## Results

Study sample characteristics are presented in Table 1. In total, 52.5% were women. Mean age was 57.2 years in women and 57.4 years in men. Prevalence of hazardous alcohol use was 5.6% in women and 18.4% in men. Insomnia prevalence was 24.1% in women and 15.0% in men. The proportions of participants with insomnia according to hazardous alcohol use and non-hazardous alcohol use are presented in Table 2. In total, 24.1% of the participants with hazardous alcohol use also reported insomnia, relative to 18.9% of the non-hazardous alcohol users (*p* < 0.001). A significantly higher proportion of women with hazardous alcohol use, reported concurrent insomnia compared to women without hazardous use (33.5% versus 23.3%, *p* < 0.001), the same was observed among men with and without hazardous alcohol use (21.1% versus 13.5%, *p* < 0.001).

**Table 1:** Sample characteristics. The Tromsø Study 2015–2016 (*N* = 19 185)

	**Women** **(***n* = 9911–10,874)^e^	**Men** (*n* = 9274–10,009)^e^
**Age, years (SD)**	57.2 (11.5)	57.4 (11.4)
**Education, %**		
Primary school	24.1 (2617)	22.2 (2179)
Secondary school	25.4 (2759)	30.5 (2997)
University/college < 4 years	17.6 (1917)	21.3 (2091)
University/college > 4 years	32.9 (3581)	26.1 (2564)
**Live with spouse, %**	72.3 (7403)	81.6 (7880)
**Work shifts, %**	9.3 (976)	11.5 (1102)
**Hazardous alcohol use**^a,^ **%**	5.6 (551)	18.4 (1710)
**Insomnia (DSM-5)**^b,^ **%**	24.1 (2567)	15.0 (1455)
**Somatic disease**^c,^ **%**	64.2 (7073)	58.4 (5830)
**Sleep medication**		
Not used	88.1 (9201)	94.1 (9099)
Less frequently than every week	5.4 (564)	2.9 (282)
Every week, but not daily	3.5 (370)	1.5 (148)
Daily	2.9 (305)	1.4 (139)
**Anxiolytic medication**		
Not used	96.0 (9898)	97.7 (9398)
Less frequently than every week	2.1 (213)	1.1 (101)
Every week, but not daily	0.9 (91)	0.5 (48)
Daily	1.1 (113)	0.8 (72)
**Antidepressant medication**		
Not used	96.0 (9885)	98.0 (9401)
Less frequently than every week	0.5 (53)	0.3 (27)
Every week, but not daily	0.3 (26)	0.2 (16)
Daily	3.2 (329)	1.59 (153)
**Mental distress**^d,^ **mean (SD)**	1.35 (0.41)	1.24 (0.35)

Numbers are means for continues variables (standard deviation) and proportion (number) for categorical variables

^a^ Hazardous alcohol use was defined by an AUDIT score of > 8

^b^ Insomnia was defined as scoring > 3 days on sleep onset latency, sleep maintenance or early morning awakening and > 3 days on either daytime impairment or dissatisfaction, a duration criteria of > 3 months was set in accordance with the DSM-5 criteria for insomnia

^c^ Somatic disease was defined as a positive response to one of the following diseases: hypertension, myocardial infarction, heart failure, arterial fibrillation, angina pectoris, stroke, diabetes, kidney disease, chronic pulmonary disease, asthma, cancer, arthritis, arthrosis and migraine, past or present

^d^ Mental distress was mean scored, range 1–4

^e^ n was lowest for hazardous alcohol use, and highest for age

**Table 2:** Prevalence of insomnia without or with hazardous alcohol use. The Tromsø Study 2015–2016 (*N* = 18 898)

	**No hazardous alcohol use**^**a**^			**Hazardous alcohol use**^**b**^			
	% (n)	95% CI	SE	% (n)	95% CI	SE	*p*
Women	23.3 (2137)	22.4, 24.1	.004	33.5 (183)	29.5, 37.4	.020	< .001*
Men	13.5 (1004)	12.7, 14.2	.004	21.1 (359)	19.1, 23.0	.010	< .001*
Total	18.9 (3141)	18.2, 19.5	.003	24.1 (542)	22.3, 25.9	.009	< .001*

^a^ Insomnia was defined as scoring > 3 days on sleep onset latency, sleep maintenance or early morning awakening and > 3 days on either daytime impairment or dissatisfaction, a duration criteria of > 3 months was set in accordance with the DSM-5 criteria for insomnia

^b^ Hazardous alcohol use was defined by an AUDIT score of > 8

The 4554 participants excluded from the multivariate analysis had a lower prevalence of hazardous alcohol use compared to the 16 529 included for analysis (10.3% versus 12.4%) which was statistically significant x^2^(1.17754)8.03, *p* = 0.005. They had a significantly higher prevalence of insomnia compared to included participants (18.6% versus 17.6). In addition, they had a statistically higher mean value of mental distress (1.34 versus 1.28) than the 16 529 who were included for analysis, *t*(20460)9.11, *p* < 0.001. Excluded participants were also significantly more likely to be female (x^2^(1,21083) 225.4, *p* < 0.001) and had a higher mean age than included participants (62.2 versus 56.0 years) *t*(21081)33.7, *p* < 0.001.

The nested logistic regression analysis showed a significant relationship between hazardous alcohol use and insomnia. The interaction term (hazardous alcohol use x gender) did not significantly modify this relationship, neither did adjustment for sociodemographic and socioeconomic factors, or somatic disease and use of psychopharmacological drugs. However, in the fully adjusted model that included mental distress, the relationship between hazardous alcohol use and insomnia was non-significant (see Table 3). Being female, having lower education, working shifts, not living with a spouse, having somatic health problems and using sleep medication were also independently associated with higher odds of having insomnia.

**Table 3:** Predictors of insomnia by logistic regression analysis. The Tromsø Study (2015–2016) (*N* = 16 529)

	**Model 1**^a^		**Model 2**^b^		**Model 3**^c^		**Model 4**^d^	
	**Odds ratio**^a^ **95% (CI)**	***p***	**Odds ratio (95% CI)**	***p***	**Odds ratio (95% CI)**	***p***	**Odds ratio (95% CI)**	***p***
Hazardous alcohol use	1.64 (1.34. 2.01)	< .001	1.66 (1.36, 2.03)	< .001	1.49(1.20, 1.85)	< .001	1.00 (.78, 1.27)	.975
Sex (women = 0)	.51 (.47, .56)	< .001	.51 (.47, 56)	< .001	.58 (.53, .64)	< .001	.71 (.64, .78)	< .001
Sex x hazardous alcohol use ^e^	1.04 (.81, 1.33)	.752	1.01 (.79, 1.30)	.935	1.02 (.79, 1.3)	.884	1.09 (.82, 1.46)	.552
Age			1.00 (1.00, .1.00)	.549	.99 (.98, .99)	< .001	1.01 (1.00, 1.01)	.027
Education (lowest = 1, highest = 4)			.91 (.88, .95)	< .001	.93 (.89, .96)	< .001	.95 (.91, .99)	.011
Live with spouse (no = 0)			.77 (.70, .84)	< .001	.84 (.76, .93)	< .001	.93 (.84, 1.04)	.199
Working shifts (no = 0)			1.22 (1.08, 1.39)	.002	1.24 (1.09, 1.41)	< .001	1.32 (1.14, 1.51)	< . 001
Somatic disease (0 = no)					1.57 (1.43, 1.71)	< .001	1.35 (1.23, 1.49)	< .001
Sleep medication					2.73 (2.52, 2.95)	< .001	2.22 (2.05, 2.42)	< .001
Tranquilizing medication					1.07 (.93, 1.23)	.338	.77 (.66, .90)	< .001
Antidepressant medication					1.06 (.96, 1.16)	.239	.76 (.69, .85)	< .001
Mental distress							10.93 (9.65, 12.36)	< .001

^a^ *OR* Odds ratio. *CI* confidence interval

^b^ Model 1 included hazardous alcohol use, sex, and the sex interaction term

^c^ In model 2, education level, marital status and whether respondents worked shifts were included

^d^ Model 3 included somatic disease, use of sleep, tranquilizing or antidepressant medication

^e^ In the fully adjusted model (Model 4), mental distress was included

^f^ Interaction between sex and hazardous alcohol use

**Fig. 1:**
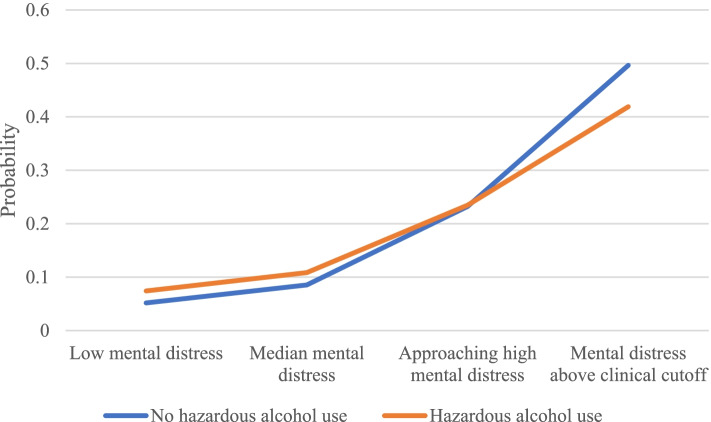
The probability of insomnia depending on hazardous alcohol use and levels of mental distress (*N* = 16 529). The moderator ‘mental distress’ was defined according to the cut-offs used when probing the interaction and identified as the regions of significance using the Johnson-Neyman technique. Using a non-centered mental distress variable these regions correspond to the following cut off values for mental distress, i.e. up to 1 (*n* = 5 494), from 1.1–1.20 (*n* = 4 594), from 1.21–1.60 (*n* = 4 265), from 1.61 up to 1.90 (*n* = 1 137) and 1.91 and above (*n* = 1 039)

In the moderation analysis including the term mental distress × hazardous alcohol use, the conditional effect of hazardous alcohol use on insomnia (log odds = 0.19/OR = 1.20, S.E. = 0.08, *p* < 0.014) was significant, as was the conditional effect of mental distress (log odds = 2.50/OR = 13.40, S.E. = 0.07, *p* < 0.001). The moderation effect was also significant (log odds = -0.58,/OR = 0.56, S.E. = 0.14, *p* < 0.001). Probing the interaction showed that hazardous alcohol use was positively associated with insomnia, but only for individuals with low (16^th^ percentile; log odds = 0.35/OR = 1.98, SE = 0.10, *p* < 0.001) or median levels (50^th^ percentile; log odds = 0.23/OR = 1.26, SE = 0.08, *p* = 0.004) of mental distress. For individuals approaching high levels of mental distress, hazardous alcohol use was not significantly associated with insomnia (84^th^ percentile; log odds = 0.00/OR = 1.00, SE = 0.07, *p* = 0.991). However, the Johnson-Neyman regions of significance showed two values in which the conditional effect of hazardous alcohol use on insomnia became significant, i.e. mental distress $\le .08$ and $\ge .62$. The lower region (values $\le .08$ on mental distress) corresponds to the significant conditional effect of hazardous alcohol use on insomnia for low and approximating median levels of mental distress. The higher region (values $\ge .62$ on mental distress) corresponds to the conditional effect of hazardous alcohol use on insomnia at very high levels of mental distress, or mental distress above the established cut-off for identification of persons with a likely anxiety or depression diagnosis [33]. Only 6.29% of the sample scored $\ge .62$ on mental distress. Thus, this result makes it evident that the conditional effect of hazardous alcohol use on insomnia at very high levels of mental distress is negative, and significant, indicating that for individuals with high levels of mental distress ($\ge .62$) the odds of having insomnia were lower than for individuals with a non-hazardous alcohol use. The moderating effect is displayed in Fig. 1. The conditional effects of hazardous alcohol use at different values of the moderator are displayed in supplementary Table 1.

## Discussion

In this population-based study, the main finding was the higher prevalence of insomnia among participants with a hazardous alcohol use. However, the moderating effect of mental distress showed that hazardous alcohol use is related to insomnia only at low or medium levels of mental distress, which may indicate that mental distress may be a more important predictor for insomnia.

The higher prevalence of insomnia among participants with hazardous alcohol use are in line with previous findings [5, 18, 23]. Our findings are in the lower end of the range compared to epidemiological studies from the US [22, 23], which may be explained by the focus upon more severe alcohol problems in these studies. Our findings are comparable to a study from Sweden [18], except that it only included men, implicating that their estimates may have been somewhat higher had they included women. Thus, it is difficult to compare our results with other epidemiological studies, due to methodological differences.

More clinically severe alcohol problems may be related to an even higher probability of insomnia [23]. However, as alcohol problems occur along a spectrum of severity, it is equally important to estimate the probability at lower ends of the spectrum that in turn may progress in to more serious alcohol problems over time [9]. A potential explanation for the relationship between hazardous alcohol use and insomnia, is the neurochemical effect of alcohol on sleep, disrupting REM sleep in the second half of the night [35], a period associated with the greatest physiological recovery [35].

Men in this study had higher proportion rates of hazardous alcohol use than women, and women reported higher levels of insomnia compared to men. These findings are consistent with some previous findings [5, 23], and a meta-analysis concluding that women have a higher predisposition for insomnia compared to men [7]. It is, however, unclear whether this is due to affective disorders known to appear highly comorbid with insomnia, or potential gender differences in sleep physiology [7]. However, the relationship between hazardous alcohol use and insomnia did not differ for men and women, as indicated by the non-significant moderating effect of sex in the logistic regression analyses.

We found hazardous alcohol use to be associated with increased insomnia, however, the inclusion of mental distress rendered this relationship non-significant. This finding contradicts two prospective population studies conducted in the US [23, 36] where alcohol dependence remained a risk factor also after adjustment for a history of mental health conditions (affective, anxiety, psychotic and drug use disorders). Moreover, some previous studies have not adjusted for mental distress [5, 17, 37], which may inflate the statistical relationship between alcohol use and insomnia. Anxiety and depression are the disorders most often occurring comorbid with alcohol use disorders and insomnia disorders [38] and there may be a causal relationship to both alcohol use [39] and insomnia [40]. The fact that an association between insomnia and hazardous alcohol use was present only at low to moderate levels of mental distress may support a self-medication hypothesis which has been suggested in the literature [42–44]. The sedative effect of alcohol [35] may have short term mood-altering effects, thereby temporarily numb the mental distress, and hence decrease insomnia symptoms. The group scoring above cut-off for hazardous alcohol use had lower odds of insomnia at high levels of mental distress in the moderation analysis in the current study. This may indicate that for some, their high burden of anxiety and depression consuming alcohol at hazardous levels do not add to the total burden towards experiencing insomnia. Whereas for participants with non-hazardous alcohol use, approaching high levels of mental distress yielded a higher probability of insomnia. This may suggest that those with hazardous alcohol use may experience a self-medicative effect from consuming alcohol in the association with insomnia. Supporting this hypothesis, a qualitative study from the UK found that the main reasons for using alcohol or other substances to self-medicate, was to lower symptoms of anxiety, depression and sleeplessness [41]. Also, ethanol has been found to acutely impact both sleep and mood of individuals with insomnia to a greater degree than normal sleepers, thus reinforcing the usage of alcohol as a hypnotic and mood altering substance specifically for individuals struggling with sleep [42].

Although a causal relationship cannot be inferred from the cross-sectional associations identified in the present study, the validity of our findings draw support from studies highlighting the high comorbidity of hazardous alcohol use, insomnia and mental health problems, in particular anxiety and depression [43].

### Strengths and limitation

A strength of this study is the large sample with equal proportions of women and men from a general population with a reasonably high attendance. Selection bias however, cannot be ruled out. Participants excluded  from the multivariate analysis in the present study had a lower prevalence of hazardous alcohol use, higher prevalence of insomnia, and a higher mean score of mental distress compared to participants included for analysis. Furthermore, excluded participants were more likely to have a higher mean age, and to be women, compared to the participants included. Although we did not have data on those who did not consent to participate in the seventh survey of the Tromsø Study, another study examined this and found nonattenders to be women, and to have a higher mean age compared to attenders [44] which is in line with the participants excluded from analysis in the current study. In addition, another Norwegian population-based study [45] found that nonattenders were more likely to suffer from psychiatric illness, compared to attenders. Also, since the seventh survey of the Tromsø Study only included women and men aged > 40 + , our findings are not generalizable for a younger population. The use of validated instruments for hazardous alcohol use, insomnia, and mental distress provides more precise estimates than using only single question items. Our estimates were also adjusted for several potential confounders, allowing for higher precision. A possible limitation is the use of same cut-off score for AUDIT for women and men, instead of a lower score for women [9]. However, using this cut-off yielded the same trend in prevalence of hazardous alcohol use as found in a Norwegian epidemiologic study by Kringlen and colleagues [46]. In case a lower cut-off score would have been more accurate, the result would be a higher prevalence of insomnia among women with hazardous alcohol use, indicating that our estimates may be somewhat low. Furthermore, the general low cut-off score on the AUDIT yielded a high overall prevalence of hazardous alcohol use, on the less severe end of the spectrum of alcohol problems. Thus, the results of even low-level alcohol issues are associated with sleep problems, implying that our findings can be applied to a broader population.

## Conclusion

The findings from this general population sample showed that having a hazardous alcohol use yielded a higher prevalence of insomnia. However, the presence of mental distress was more crucial for the increased probability of insomnia. This needs to be taken into account when analyzing associations between hazardous alcohol use and insomnia problems. The findings in the present study underline the importance of screening for hazardous alcohol use and mental distress among patients presenting with insomnia in primary care as these are common comorbid conditions, and hazardous alcohol use and mental distress may be a contributing factor to insomnia. This may potentially result in severe consequences. Future research could benefit from a longitudinal design to further investigate the role of mental distress on alcohol use and insomnia over time in order to study causal inference.

## Supplementary Information

**Additional file 1: Supplementary Table 1.** Conditional effects of hazardous alcohol use at different values of the moderator, mean centered mental distress. The Tromsø Study (2016-2015).

## Data Availability

The datasets generated and/or analysed during the current study are not publicly available as they came from a third party. Legal restrictions protect against potential reverse identification and of de-identified participant information. The data can be made available upon application to the Tromsø Study Data and Publication Committee (The Tromsø Study, Department of Community Medicine, Faculty of Health Sciences, UiT The Arctic University of Norway), email: tromsous@uit.no.

## Abbreviations

AUDIT: Alcohol Use Disorder Identification Test
BIS: Bergen Insomnia Scale
DSM-5: Diagnostic and Statistical Manual of Mental Disorders 5^th^ edition
EM: Expectation Maximization
HSCL-10: Hopkins Symptoms Checklist 10
ICD-10: International classification of Diseases 10^th^ edition
MVA: Missing Values Analysis
REM: Rapid Eye Movement
