# Supplementary material for: Epidemiology of comorbid hazardous alcohol use and insomnia in 19 185 women and men attending the population-based Tromsø Study 2015–2016
Source: BMC Public Health. 2022 Apr 27;22:844. doi: 10.1186/s12889-022-13250-5 (PMC9047295; doi:10.1186/s12889-022-13250-5)
Supplement: Supplementary file 1 — Additional file 1: Supplementary Table 1. Conditional effects of hazardous alcohol use at different values of the moderator, mean centered mental distress. The Tromsø Study (2016-2015). [file 12889_2022_13250_MOESM1_ESM.docx]

| Values of mental distress | Log odds | *CI* | *p.* |
| --- | --- | --- | --- |
| -.28 | .35 | .16, .55 | <.001 |
| -.14 | .26 | .10, .43 | .002 |
| .01 | .18 | .03, .32 | .017 |
| .08 | .14 | .00, .28 | .050 |
| .16 | .09 | -.05, .22 | .193 |
| .31 | .01 | -.14, .14 | .980 |
| .46 | -.09 | -.24, 07 | .268 |
| .61 | -.17 | -.35, .00 | .054 |
| .62 | -.18 | -.35, .00 | .050 |
| .76 | -.26 | -.47, -.05 | .013 |
| .91 | -.35 | -.59, -.11 | .005 |
| 1.06 | -.44 | -.71, -.16 | .002 |
| 1.21 | -.52 | -.84, -.21 | .001 |
| 1.36 | -.61 | -.97, -.26 | <.001 |
| 1.52 | -.70 | -1.10, -.30 | <.001 |
| 1.67 | -.79 | -1.22, -.35 | <.001 |
| 1.82 | -.87 | -1.35, -.40 | <.001 |
| 1.97 | -.96 | -1.48, -.44 | <.001 |
| 2.12 | -1.05 | -1.61, .49 | <.001 |
| 2.27 | -1.14 | -1.74, -.53 | <.001 |
| 2.42 | -1.23 | -1.87, -.58 | <.001 |
| 2.57 | -1.31 | -2.00, -.63 | <.001 |
| 2.72 | -1.40 | -2.13, -.67 | <.001 |
| **Supplementary Table 1.** Conditional effects of hazardous alcohol use at different values of the moderator, mean centered mental distress. The Tromsø Study (2016-2015). | | | |
|  | | | |
